# Bridging the Bridging Imidazolate in the Bimetallic Center of the Cu/Zn SOD1 and ALS

**DOI:** 10.3389/fchem.2021.716438

**Published:** 2021-09-03

**Authors:** Ahmet Can Timucin, Suleyman Selim Cinaroglu, Osman Ugur Sezerman, Emel Timucin

**Affiliations:** ^1^ Department of Molecular Biology and Genetics, Acibadem MAA University, Istanbul, Turkey; ^2^ Department of Biochemistry, University of Oxford, Oxford, United Kingdom; ^3^ Department of Medical Informatics and Biostatistics, School of Medicine, Acibadem MAA University, Istanbul, Turkey

**Keywords:** superoxide dismutase 1, metal binding, molecular dynamics simulation, stabiliy, dimer

## Abstract

Metallation status of human Cu/Zn superoxide dismutase 1 (SOD1) plays a pivotal role in the pathogenesis of amyotrophic lateral sclerosis (ALS). All of the amino acids found in the bimetallic center have been associated with ALS except for two positions. H63 which forms the bridging imidazolate ion in the bimetallic center and K136 which is not directly involved in coordination but located in the bimetallic center were not reported to be mutated in any of the identified ALS cases. In this study, we investigated the structure and flexibility of five SOD1 variants by using classical molecular dynamics simulations. These variants include three substitutions on the non-ALS-linked positions; H63A, H63R, K136A and ALS-linked positions; G37R, H46R/H48D. We have generated four systems for each variant differing in metallation and presence of the intramolecular disulfide bond. Overall, a total of 24 different dimers including the wild-type were generated and simulated at two temperatures, 298 and 400 K. We have monitored backbone mobility, fluctuations and compactness of the dimer structures to assess whether the hypothetical mutations would behave similar to the ALS-linked variants. Results showed that particularly two mutants, H63R and K136A, drastically affected the dimer dynamics by increasing the fluctuations of the metal binding loops compared with the control mutations. Further, these variants resulted in demetallation of the dimers, highlighting probable ALS toxicity that could be elicited by the SOD1 variants of H63R and K136A. Overall, this study bridges two putative SOD1 positions in the metallic center and ALS, underlining the potential use of atomistic simulations for studying disease variants.

## 1 Introduction

Amyotrophic lateral sclerosis (ALS) which is characterized by degeneration of upper and lower motor neurons (Rowland and Shneider, 2001) is the most common late-onset motor neuron disease (Renton et al., 2014). This disease’s first leap into public consciousness was occurred when it afflicted the athlete named Lou Gehrig who had succumbed to death 2 years after his diagnosis (Ray and Lansbury, 2004). Despite the scientific progress made ever since, its underlying toxicity mechanism still needs to be addressed at the molecular level in order to develop effective therapeutic strategies against ALS (Zou et al., 2016; Chia et al., 2018; Huai and Zhang, 2019).

Akin to most neuro-degenerative diseases, ALS can present itself in both sporadic and familial forms. Although the etiology of the sporadic form remains largely elusive, the familial form (fALS) has been linked to genetic mutations in the coding regions of more than 20 genes (Li and Wu, 2016). In fact, this form of ALS has been first established by identification of the mutations in the free radical scavenging enzyme Cu/Zn superoxide dismutase 1 (SOD1) (Rosen et al., 1993). Since then, more than 200 SOD1 mutations have been identified (https://alsod.ac.uk/) accounting for 20–25% of fALS and ∼6% of all ALS cases (Pasinelli and Brown, 2006; Rotunno and Bosco, 2013).

**FIGURE 1:**
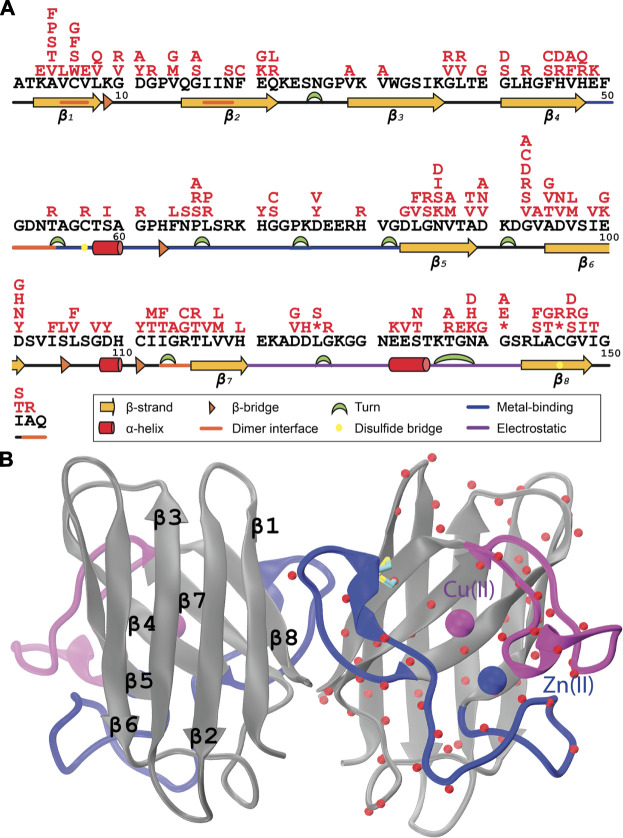
**(A)** ALS-associated mutations (red) were shown on the SOD1 protein sequence (black), **(B)** C*α* atoms of the mutated positions were illustrated on one of the subunits of the human SOD1 dimer (PDB ID: 2V0A) (Strange et al., 2007). Metal-binding and electrostatic loops were colored in blue and purple for each subplot, respectively.

A large portion of the ALS-linked SOD1 mutations are missense mutations while only one fifth of the variations are due to insertions and/or deletions or nonsense mutations (Rotunno and Bosco, 2013). Essentially, ALS-causing missense mutations were not confined to any particular regions of SOD1, instead they have been widely scattered over the sequence (Figure 1A) and structure (Figure 1B). Furthermore, most of the mutations are conservative replacements that are expected to impose only subtle alterations to the structure and function. Given the pattern-less spread of mutations over SOD1 and dominance of conservative substitutions, deciphering a unifying toxicity mechanism elicited by SOD1 in ALS has become a challenging task, increasing the attention towards SOD1 structure.

**FIGURE 2:**
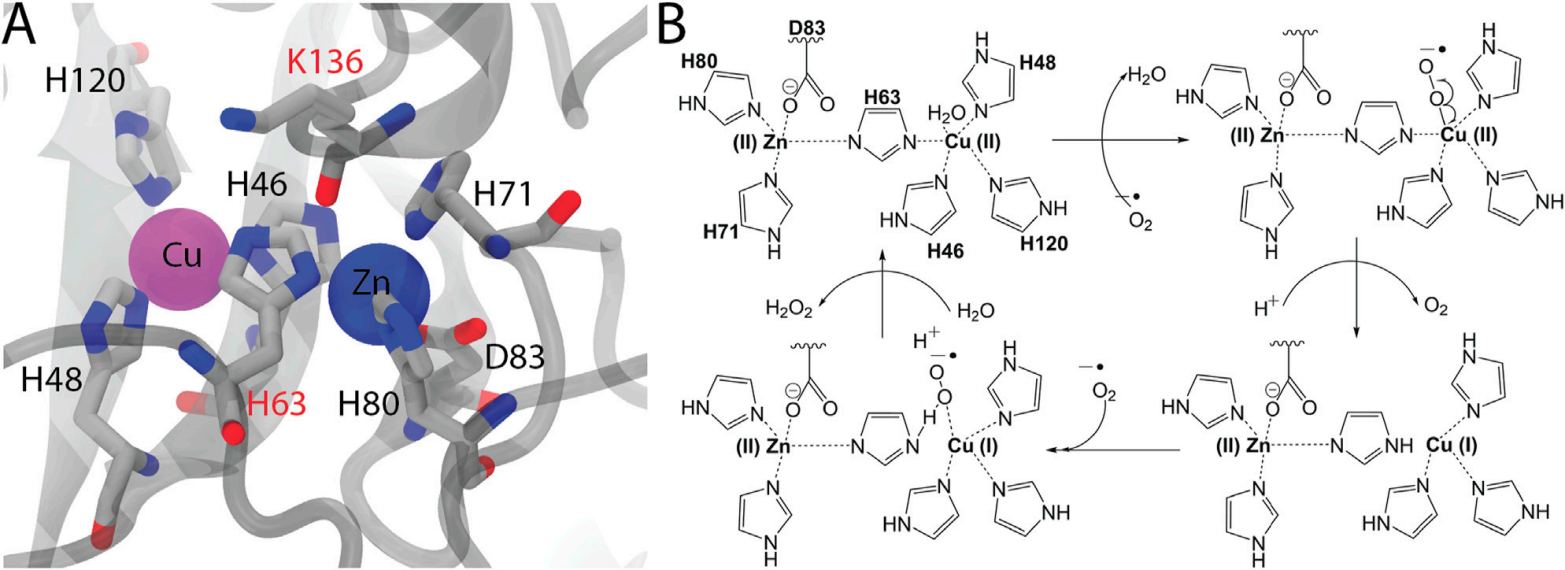
**(A)** The close-up view of the bimetallic center and surrounding amino acids that are located in the first coordination shell (<5 Å) were visualized in the 2V0A structure (C: cyan, O: red, N: blue). The amino acids that were not linked to any ALS cases were shown by red-labels. **(B)** Proposed mechanism of the SOD1 reaction wherein Cu-center acts as the catalytic center.

Human Cu/Zn SOD1 gene encodes a highly conserved (Fridovich, 1995) 153-aa long protein which folds into a well-studied 8-stranded *β*-barrel structure (Figure 1B) from the immunoglobulin fold family (Richardson et al., 1976). This barrel structure is flanked by two large loops connecting the strands of *β*4-*β*5 and *β*7-*β*8 that hold the Cu/Zn bimetallic center (Figure 1B). The Cu/Zn bimetalic center in SOD1 features a striking bridging histidine in the imidazolate tautomeric form (Strothkamp and Lippard, 1982). This type of histidine tautomerization was in fact an unprecedented feature for metalloproteins. In parallel with its uniqueness, the imidazolate bridged metallic center plays a pivotal role both in the stability and activity of the enzyme. Notably, this structure is one of the most stable structures (Rakhit and Chakrabartty, 2006). While multiple structural features such as dimerization and the conserved intramolecular disulfide bond between C57 and C146 (Figure 1B) were shown to contribute to the extreme stability of SOD1 (Furukawa and O’Halloran, 2005; Goodsell and Olson, 2000), the imidazolate-bridged metallic center was also associated with the structural stability of the enzyme (Arnesano et al., 2004). Essentially, the fully metallated SOD1 can tolerate extreme conditions such as elevated temperatures and denaturating media (Lepock et al., 1985; Rakhit and Chakrabartty, 2006; Forman and Fridovich, 1973). Besides, the metallic center, explicitly the Cu^2+^ acts as the catalytic center of the dismutase reaction (Figure 2A). Due to the critical roles of the Cu/Zn center for the stability and activity of SOD1, ALS-linked mutations that affect the integrity of the metal center are exclusively studied as *metal-binding* mutants. On the other hand, the mutations that do not alter metallation status of the variants generally termed as *wild-type-like* mutants. Consistently, *metal-binding* mutants that are closely located to the bimetallic center, if not directly involved in metal coordination, were characterized by discernibly low stability and/or activity profiles while *wild-type-like* mutants did not exert significant changes in the stability or activity of the variants (Oztug Durer et al., 2009). Accordingly, SOD1 variants with a disrupted metallic center can undergo distinct conformational changes with lowered stability than the fully metallated forms leading to misfolding and aggregation (Tiwari et al., 2009; Banci et al., 2007; Ding and Dokholyan, 2008; Wright et al., 2019; Wang et al., 2002, 2003, 2007). In fact, demetallation-induced SOD1 aggregation has been widely referred as the gain-of-function mechanism behind SOD1-induced toxicity in fALS (Hilton et al., 2015). Furthermore, current therapeutic approaches in fALS aim to mediate metallation status of SOD1 variants, reflecting the significance of *metal-binding* SOD1 mutants in ALS pathology (Hilton et al., 2015; Tokuda and Furukawa, 2016). Overall, ALS-linked SOD1 mutants, particularly those affecting metallation status of the variants are of high importance for a better understanding of the SOD1-mediated toxicity in this disease.

MD simulations present alternative methodologies that can access the dynamics of macro-molecules (Adler-Abramovich et al., 2012; Mera-Adasme et al., 2013). Specifically, many studies have used MD simulations to investigate structural mechanism behind SOD1-mediated ALS toxicity (Banci et al., 1992; Strange et al., 2007; Srinivasan and Rajasekaran, 2017; Pereira et al., 2021). To elaborate the impact of the hypothetical SOD1 mutations on the dimer dynamics, we have employed classical molecular dynamics simulations to study six SOD1 variants. Two of these variants were established ALS-variants while three of them were not associated with any ALS cases (Lill et al., 2011). Nonetheless, particularly the close proximity of these positions to the bimetallic center, we surmise that their mutations would affect the metallation status of the enzyme, leading to similar toxic misfolded aggregates. Our results pointed out that essentially H63 and K136 variants behaved similar to the ALS-linked variants reflecting the potential toxicity associated with these novel positions.

## 2 Methods

### 2.1 Structure Selection

The crystal structure 2V0A (Strange et al., 2007) was recruited. This structure did not contain any mutations and captured in the dimeric form which represent the active form the wild-type SOD1 (Fridovich, 1975). We have utilized the dimeric form in our studies, which holds two metal ions. The metal ions, zinc and copper, were kept in the holo structures as $Zn^{2+}$ and $Cu^{2+}$, representing the second ground dimer Prior to simulations, multiple occupancies were eliminated from the structure. In silico mutagenesis was applied to obtain five different mutations, namely H63A, H63R, K136A, G37R and H46R/H48D. For every wild-type and mutant dimers, four distinct structures were generated: 1) apo dimer (non-metallated) with an oxidized disulfide bond (C57-C146), 2) apo dimer (non-metallated) with a reduced disulfide bond, 3) holo dimer (metallated) with an oxidized disulfide bond and 4) holo dimer (metallated) with a reduced disulfide bond. Overall, 24 different SOD1 dimers were generated. All of the structures were protonated according to pH 7.0 by PROPKA (Li et al., 2005; Søndergaard et al., 2011). Protonated structures were solvated in a cubic box of water modeled by TIP3P (Jorgensen et al., 1983). Solvated systems were neutralized by sodium and chloride counter-ions.

### 2.2 Molecular Dynamics Simulations

Resulting systems containing wild-type and SOD1 mutant enzymes were initially energy minimized by the steepest descent approach without any constraints. Molecular dynamics simulations were performed for all of the 24 dimeric SOD1 by using Gromacs 5.1.4 (Berendsen et al., 1995; Van Der Spoel et al., 2005; Pronk et al., 2013; Abraham et al., 2015) and adopting the CHARMM36 force field Phillips et al. (2005); MacKerell and Bashford, (1998); Brooks et al. (2009); Huang and MacKerell (2013). CHARMM was previously used for the SOD1 dimer producing results that were well aligned with experiments (Strange et al., 2007). Particle Mesh Ewald (PME) method was used for the electrostatic energy calculation (Darden et al., 1993). The time step was kept at 2 fs for all simulations. A cut-off distance of 10 Å was implemented for prediction the short-range interactions. Initially, the systems were slowly heated to the temperature of 298 K over 100 ps After stabilization of the system temperature at 298 K, an additional 100 ps of simulation was performed by using NPT ensembles at the constant pressure of 1 bar. The productions simulations were also run using NPT ensembles and at two different temperatures 298 and 400 K for 100 ns Systems were directly heated to 400 K. Overall 48 different MD simulations were performed. Trajectory analysis were done by monitoring backbone displacements and fluctuations, radius of gyration (*R*_*G*_) of the dimer and metal coordination states of both monomers. VMD was used for visual inspections of the structures and trajectories (Humphrey et al., 1996).

## 3 Results and Discussion

### 3.1 Selection Rationale of the Superoxide Dismutase 1 Variants

To date, 217 mutations in the coding region of SOD1 gene have been identified to be associated with ALS (https://alsod.ac.uk/) (Lill et al., 2011). A large portion of these mutations (184/217) are missense mutations which have been scattered throughout the SOD1 sequence (Figure 1A) and structure (Figure 1B). Many studies on SOD1 variants have converged that demetallation of SOD1 structure can significantly alter stability of the enzyme inducing formation of misfolded neurotoxic aggregates (Tiwari et al., 2009; Banci et al., 2007; Ding and Dokholyan, 2008; Wright et al., 2019; Wang et al., 2002, 2003, 2007). From this respect, metal-binding SOD1 mutations that directly affect the stability of the enzyme have been particularly well-studied in the aim of understanding the SOD1 toxicity in ALS (Wright et al., 2019).

Except for the H63 which acts as the bridging ion between metal ions, all of the amino acids that are directly involved in metal coordination; H46, H48 and H120 for Cu^2+^ and H71, H80 and D120 for Zn^2+^, were mutated at least in one ALS case (Figure 2A). H63 in the imidazolate form coordinates both of the metals, Cu^2+^ or Zn^2+^, through its *δ*N and *ϵ*N respectively. Despite its potential contribution to the integrity of both of the metallic centers, H63 was not mutated in any of the ALS cases documented so far (Abel et al., 2012). Furthermore, K136 which is not directly involved in metal coordination but found in the first shell (<5Å) was not linked to ALS. Similar to H63, K136 also is located at the center of the bimetallic center (Figure 2A). Along with H63, another bridge in the second shell, D124 exists forming hydrogen bonds with the H46 of Cu-shell and H71 of Zn-shell. D124 was previously observed to be mutated in ALS (Figure 1A) (Mera-Adasme et al., 2013). While all amino acids that were directly involved in metal coordination of the Cu/Zn SOD1 have been reported to be somehow associated with ALS, two amino acids, H63 and K136, that are located in the fist shell of the bimetallic center have not been linked to this disease. Hence despite the absence of epidemiological evidence bridging these positions with ALS, we appraise that any substitutions at these positions have the potential to affect the metallation of the enzyme leading to misfolding, in the same manner other metal binding mutants did. A similar prediction has been made for another metal binding mutant, H80R, for which compelling *in vitro* evidence suggesting a dramatic effect on the metalation and stability of SOD1 came first and clinical findings bridging this mutation to ALS was established, then (Wright et al., 2019).

Here, we have investigated these two non-ALS linked positions in three variants by MD simulations. We have also included a double mutant of *metal-binding*, H46R/H48D and a *wild-type-like* mutant, G37R, in our simulations. Particularly, the mutant, H46R/H48D that was mutated in two of the Cu-binding positions, has been expected to lose its metals, particularly Cu, parallel to previous findings on the Cu-binding variants (Winkler et al., 2009; Lelie et al., 2011). G37R, on the other hand, was not classified as a metal-binding mutant as G37 has been located far away from the bimetallic center (Lynch et al., 2004). Nevertheless, investigations of G37R SOD1 suggested altered metallation in this variant (Milardi et al., 2010). Hence, alongside with the wild-type SOD1, overall six SOD1 variants were recruited to our study to test whether the putative variants show similar structural dynamics to any of these ALS-linked mutants.

### 3.2 Modeling the Bimetalic Center

Cu/Zn SOD1 catalyzes the conversion of two superoxide radical ions, $O_{2}^{.-}$ to hydrogen peroxide, *H*_2_ *O*_2_ and molecular oxygen, *O*_2_ (Figure 2B). This reaction is a disproportionation (dismutation) reaction in which simultaneous oxidation and reduction of the $O_{2}^{.-}$ radical take place by the active site of the Cu-center (Pelmenschikov and Siegbahn, 2005). According to the proposed mechanism (Figure 2B), the first enzyme-substrate (ES) complex is transformed into the first intermediate by the binding of one of $O_{2}^{.-}$ to the Cu(II) center. This intermediate is then broken down to the second ES complex through a homolytic cleavage of the Cu-O bond, Cu(II) center is reduced to Cu(I), $O_{2}^{.-}$ is oxidized to *O*_2_ and H63 loses its contact with the Cu-center gaining a proton at its *δ*N. The second ES complex is formed through binding of the reduced Cu (I) center to a second $O_{2}^{.-}$. In the final step, the Cu (I) center catalyzes the reduction of $O_{2}^{.-}$ to *H*_2_ *O*_2_ transforming back into the first ES complex.

Given this reaction mechanism (Figure 2B), we stress the importance of the accurate force field parameters for the unique imidazolate-bridged bimetallic center. For this purpose, a number of studies have reported *ab initio* parameters of the Cu/Zn center by quantum mechanical (QM) calculations (Shen et al., 1990; Strange et al., 2007; Peng et al., 2018). Otherwise standard force fields do not provide the parameters for this bimetallic center particularly for the deprotonated H63 (Vanommeslaeghe et al., 2010). Here we defined the SOD1 variants through the CHARMM force field (Strange et al., 2007) but did not include the *ab initio* parameters for the metallic center (Shen et al., 1990; Strange et al., 2007; Peng et al., 2018). Because the QM parameters were developed for the wild-type metallic center (Shen et al., 1990; Strange et al., 2007; Peng et al., 2018), whilst half of the variants to be analyzed here were H63 mutants in which the imidazolate ion was deleted. Thus, we considered that usage of the first ground state of the SOD1 for all variants would lead to biased results due to incorporation of different parameters for their metallic centers, i.e. QM for the *wild-type-like* centers while standard parameters for the H63 variants. Given this caveat, we opted for the second ground state with a reduced Cu-center and a protonated H63 which can be easily defined by conventional force fields. Previous MD studies of SOD1 either did not disclose any details for the metal center parameters (Srinivasan and Rajasekaran, 2017; Pereira et al., 2021) or similar to this study modeled the SOD1 from the second ES complex (Mera-Adasme et al., 2013).

### 3.3 Backbone Mobility and Compactness of the Dimer in Superoxide Dismutase 1 Variants

**FIGURE 3:**
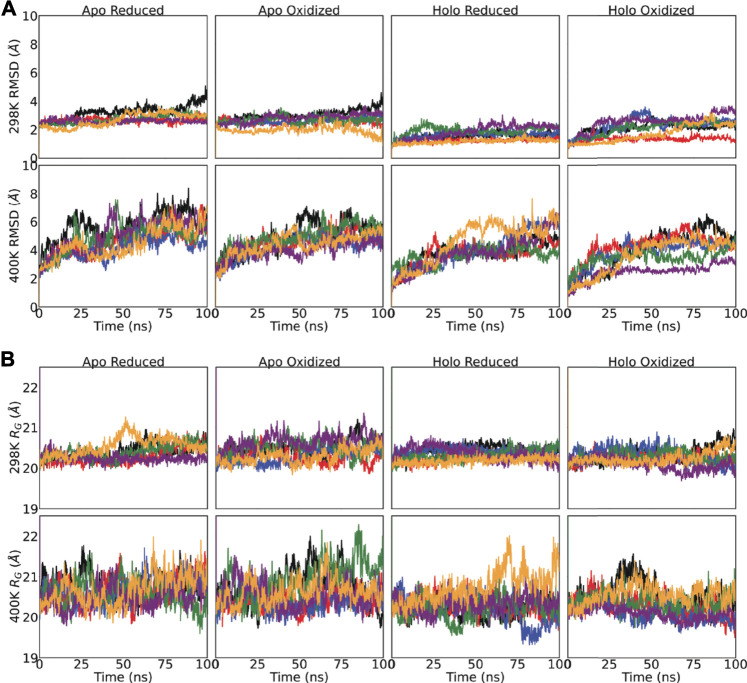
**(A)** Backbone (C*α*, C, O and N) displacement and **(B)** radius of gyration (*R*_*G*_) of the SOD1 dimers were monitored for 100 ns For both sub-plots, top-panel shows the 298 K and bottom-panel shows 400 K simulations. The same legend applies for all subplots: Wild-type-black, H63A-red, H63R-blue, K136A-green, G37R-purple, H46R/H48D-yellow.

For every SOD1 variant including the wild-type, we have generated four different structures which differed by means of the metallation and presence of the intramolecular disulfide bridge. RMSD plots show that none of the systems underwent a large structural movement regardless of the variants tested (Figure 3A). At 400 K the backbone mobility increased regardless of the metallation or disulfide bond or mutation. A similar pattern was spotted in the *R*_*G*_ of the dimer (Figure 3B). In line with RMSD measurements, the compactness of the dimer was slightly increased at 400 K for every system analyzed. On the hand, the variants, including the metal-binding double mutant, did not significantly affect the *R*_*G*_ of dimer. Overall, we have further analyzed these simulations to compare the dynamics of the hypothesised mutants with the ALS-linked variants and to elaborate the impact of metallation and disulfide bond on the dynamics of SOD1 variants.

### 3.4 Enhanced Fluctuations of the Metal Binding Loops for the H63R and K136A Variants

**FIGURE 4:**
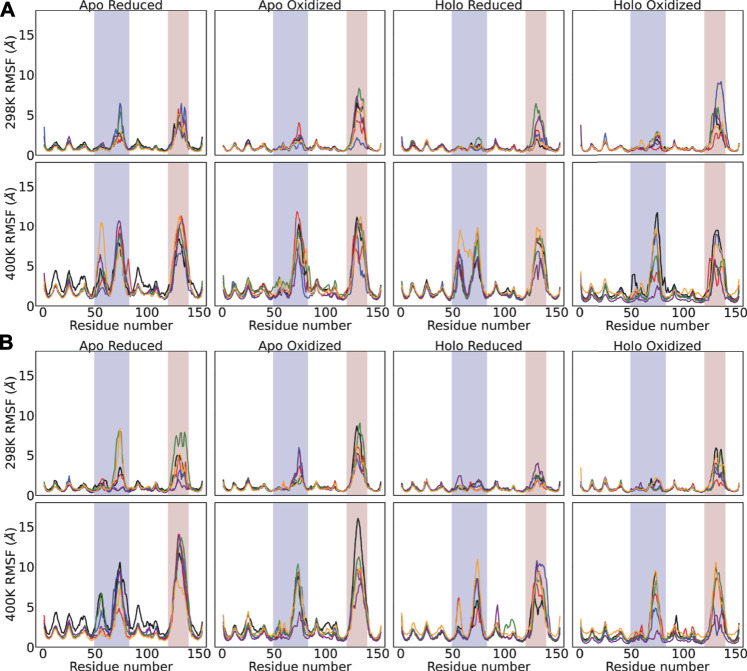
**(A)** Backbone (C*α*, C, O and N) displacement and **(B)** radius of gyration (*R*_*G*_) of the SOD1 dimers were monitored for 100 ns. For both sub-plots, top-panel shows the 298 K and bottom-panel shows 400 K simulations. The same legend applies for all subplots: Wild-type-black, H63A-red, H63R-blue, K136A-green, G37R-purple, H46R/H48D-yellow.

Fluctuations of the C*α* were monitored to compare the flexibility of the putative SOD1 variants with *metal-binding* and/or *wild-type* variants (Figure 4). One notable observation is the fact that the metal binding loops of the SOD1 structure which ligate the metals were the most flexible portion of the SOD1 structure regardless of mutation, temperature and/or disulfide bond. As such, temperature increase led to apparent increase in the flexibility of these loops which were highlighted in Figure 4. Otherwise, fluctuations of the *β*-barrel structure and termini did not significantly higher. For the wild-type enzyme we noted that the fluctuations were reduced as the structure became metalated and/or possessed an intact disulfide bond. This outcome is in line with the previous experimental observation that the stability of SOD1 structure is contributed by the disulfide bridge (Goodsell and Olson, 2000; Furukawa and O’Halloran, 2005), and the imidazolate-bridged metallic center (Arnesano et al., 2004). Among the SOD1 variants tested we noted an increase in the flexibility of the relatively shorter electrostatic loop (Figure 4). At 298 K, for the loops in the chain A (Figure 4A), the highest flexibility was observed for the H63R variant for the holo-oxidized form while for chain B (Figure 4B) the *metal-binding* double variant and K136A. These observations were also spotted in the *R*_*G*_ analyses of the loops (Supplementary Figure S1, 2). For the K136A variant, both of the loops showed reduced compactness for both the chains in the holo-oxidized form (Supplementary Figure S1), the observation which was parallel to its enhanced fluctuations (Figure 4A). Similarly, the variant H63R led to a loosened electrostatic loop particularly for the chain B (Supplementary Figure S2). On the other hand, the other variants H63A and G37R including the wild-type enzyme did not show particularly high flexibility and altered compactness compared to these three; H63R, K136A and the double mutant. When the temperature was increased to 373 K, we observed a consistent increase in the fluctuations of the metal-binding loops for every variant including the wild-type enzyme (Figures 4A,B bottom panels). Consistently the dimer compactness and flexibility have been affected implying reduced stability at high temperature. Notably, all variants including the wild-type enzyme showed enhanced fluctuations for the shorter loop reaching as high as 15 Å. Together with the compactness and fluctuation analyses, we surmise that the putative SOD1 variants could lead to altered conformations implying their potential toxicity in ALS.

### 3.5 Predicted Amyotrophic Lateral Sclerosis Toxicity for the H63R and K136A Superoxide Dismutase 1 Variants Through Cu-Depletion

**FIGURE 5:**
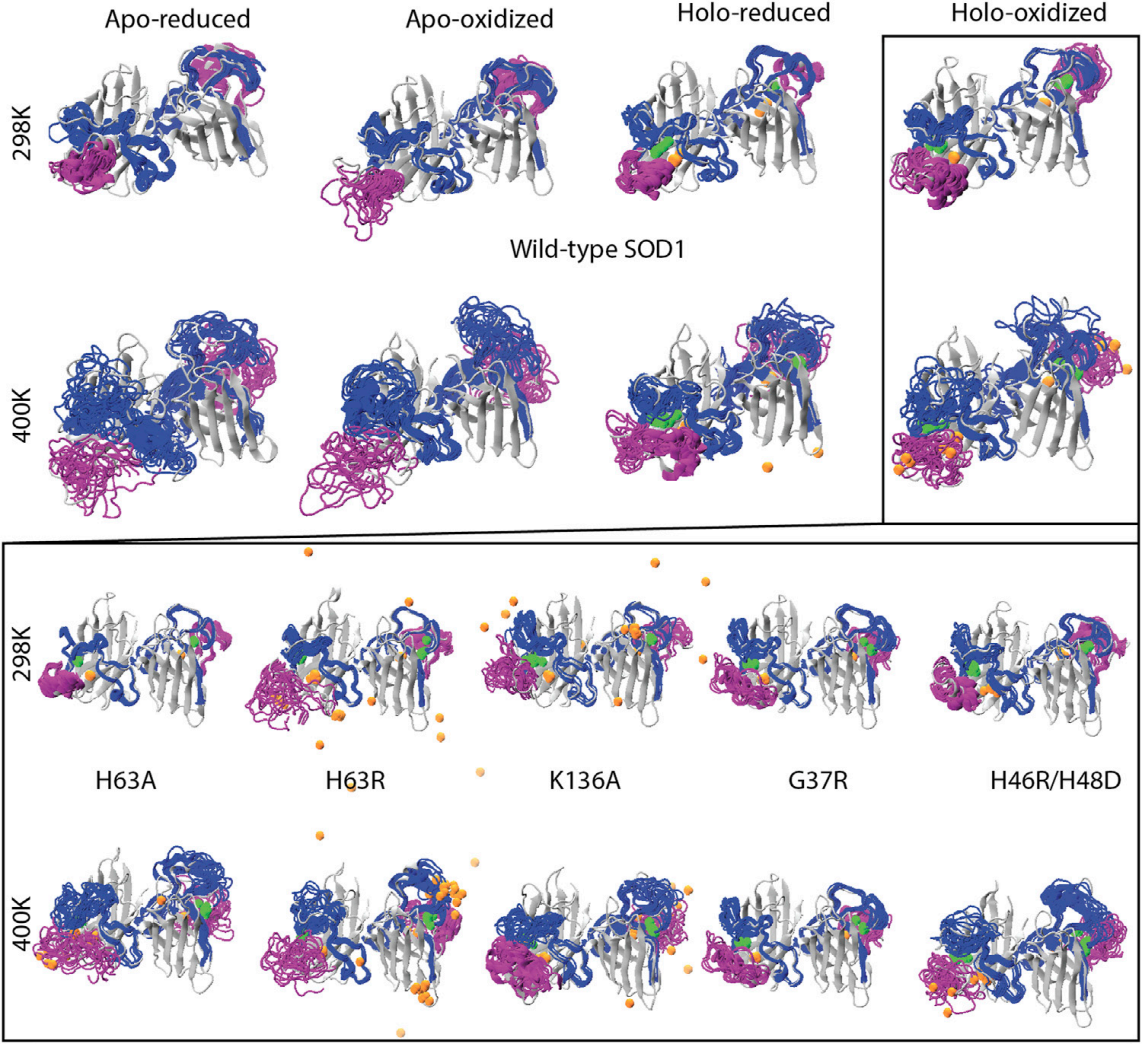
Reduced trajectories of the metal binding loops spanning the residues from 48 to 89 (blue) and from 124 to 139 (purple) were shown for the wild-type dimer in the top two panels for all four systems. Five variants in the holo-oxidized forms were similarly visualized in the bottom two panels. For all plots, Cu and Zn ions were colored orange and green respectively.

To investigate whether the SOD1 variants analyzed here restored an intact bimetallic center or not, we visually monitored the metals along with the metal-binding loops (Figure 5) and measured the average coordination distance for all systems (Supplementary Figure S3). Our results promptly suggested that the Cu-center was less tolerant to increased kinetic energy than the Zn-center (Supplementary Figure S3). This observation was aligned with the fact that Zn-center or explicitly the Zn ion has been attributed to structural stability whilst Cu ion was linked to enzymatic activity (Wright et al., 2019). At high temperature, almost all of the variants including the wild-type SOD1 showed an increase in the Cu-coordination distance with the exception of G37R (Supplementary Figure S3). This *wild-type-like* variant maintained intact Zn and Cu centers particularly when the disulfide bond was intact and less intact metal centers when the intramolecular disulfide bond was reduced. Taken together with these analyses, we pointed out that even the wild-type SOD1 and the *wild-type-like* variant G37R failed to maintain a fully intact bimetallic center, confirming that demetallation could still be the case for the wild-type or *wild-type-like* variant (Crow et al., 1997). From this point, our results were in line with the paradigm explaining altered metallation as one of the unifying toxicity mechanisms behind all ALS cases (Hilton et al., 2015).

We have visualized all of the variants in the holo-oxidized state which was shown to be have the least flexible for the wild-type (Figure 4). At 298 K we observed that Cu was released from the H63R and K136A variants while Zn was kept within the metallic center (Figure 5). Despite the increase in the average coordination distance (Supplementary Figure S3), the *metal-binding* double mutant did not particularly leave the structure. Given the increase in the average coordination distance along with the particularly increase fluctuations of the metal binding loops, we stressed that this double mutant also had an disrupted Cu-center even if Cu was not released completely. On the other hand, none of the other variants showed a particular disruption of the metal binding sites including the variant of H63A.

Overall the putative variants of H63R and K136A showed similar to or even more extreme dynamics than the *metal-binding* double mutant by means of fluctuations and metallation status. Therefore, we consider that these SOD1 variants, although they have not yet linked to ALS, could exhibit similar toxicity through demetallation. One possible explanation of why neither of these positions was not reported in any ALS cases could be due to their highly toxic nature. A similar case was noted for the *metal-binding* mutant H80R which was first generated in an animal model and its structure was shown to have a distorted Zn center due to clashes from H71 and K136 Seetharaman et al. (2010), albeit the absence of clinical evidence linking it to ALS (Wright et al., 2019). Later, a single patient who had displayed ALS symptoms at an extremely early age with a rapidly progressive disease was reported to have this mutation (Alexander et al., 2002). Hence in line with the case of H80R which has now been linked to ALS (Figure 1A), the bridging H63 and its neighbouring K136 could lead to toxic SOD1 variants leading to ALS.

Fully demetallated SOD1 variants could be targeted by the proteosomal system due to their extremely low structural stability while partially metallated variants could escape from degradation due to the fact that partial demetallation, i.e. losing one of the metals, leads to meta-stable structures (Hilton et al., 2015). Having promising therapeutic effects from both of metal-chelating and metal-delivering agents can in fact be used to support this hypothesis (Hilton et al., 2015). Accordingly, the destructive impacts of H63R and K136A on both of the metal centers resulting in the release of Cu from the structure (Figure 5) and loosing of the Zn coordination (Supplementary Figure S3) could lead to a dramatic destabilization of the structure. Thus, another plausible explanation behind lacking epidemiological data between H63R and K136A variants would lie at the other extreme. As such, these variants could be degraded by the proteosaomal system and did not include SOD1-related toxicity.

## 4 Conclusion

Identifying novel SOD1 variants related to ALS would contribute to our understanding of SOD1-mediated toxicity in ALS. In this study, we focused on the variants of two hypothetical positions. The main reason behind our particular focus on these positions is clearly their involvement in the bimetallic center in the SOD1. Given the dynamical behaviours of the H63R and K136A variants, we conclude that these SOD1 variants, although they have not yet spotted in ALS, could exhibit toxicity and thus lead to ALS.

## Data Availability

The raw data supporting the conclusion of this article will be made available by the authors, without undue reservation.
