# Supplementary material for: Bridging the Bridging Imidazolate in the Bimetallic Center of the Cu/Zn SOD1 and ALS
Source: Front Chem. 2021 Sep 3;9:716438. doi: 10.3389/fchem.2021.716438 (PMC8446448; doi:10.3389/fchem.2021.716438)
Supplement: Supplementary file 1 [file DataSheet1.PDF]

Supplementary Material

1 FIGURES

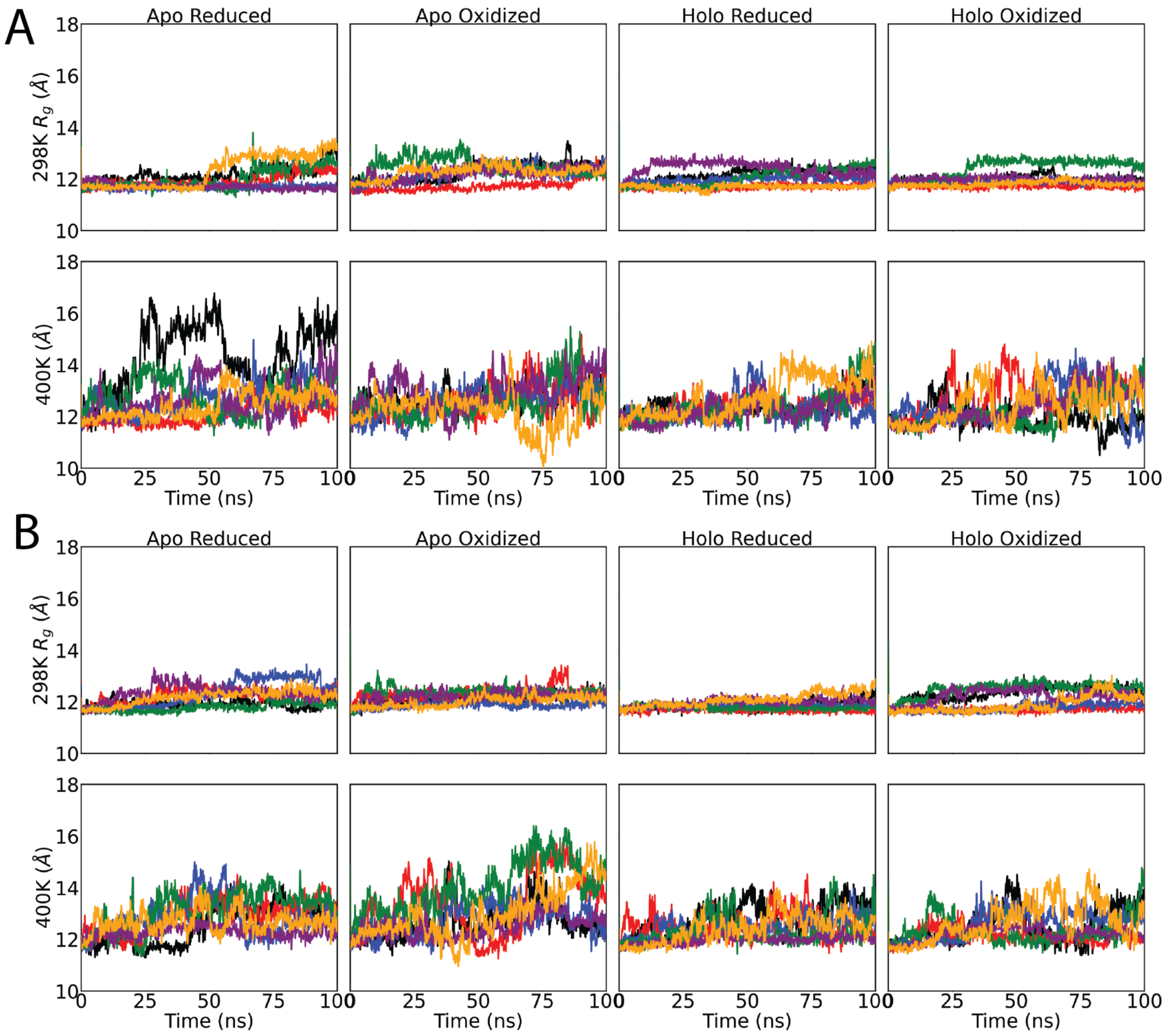

Figure S1. Radius of gyration (R_G) of the metallic-loop encompassing residues from 48 to 89 for **(A)** the chain A and **(B)** B. Wild-type-black, H63A-red, H63R-blue, K136A-green, G37R-purple, H46R/H48D-yellow.

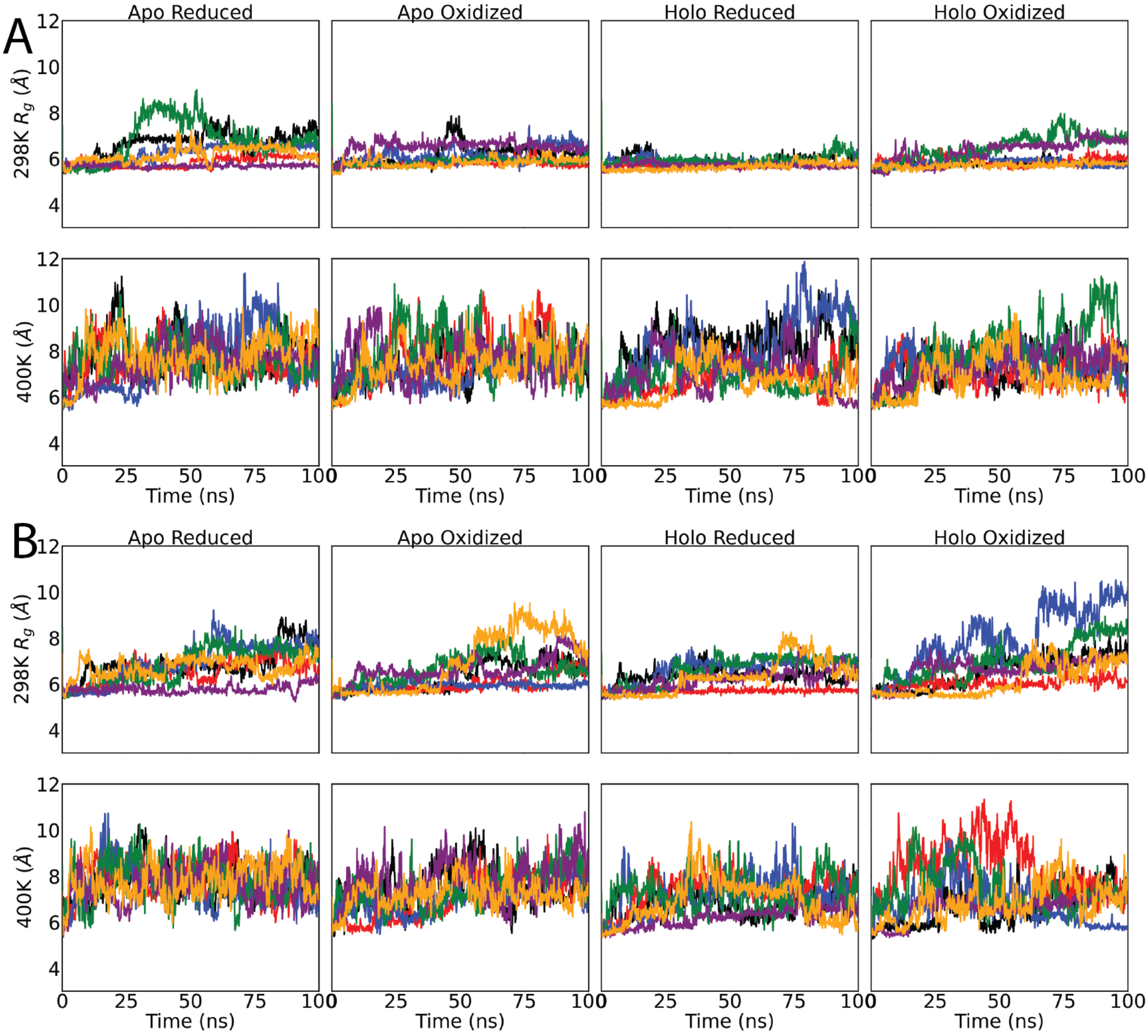

Figure S2. Radius of gyration (R_G) of the electrostatic loop encompassing residues from 124 to 139 for **(A)** the chain A and **(B)** B. Wild-type-black, H63A-red, H63R-blue, K136A-green, G37R-purple, H46R/H48D-yellow.

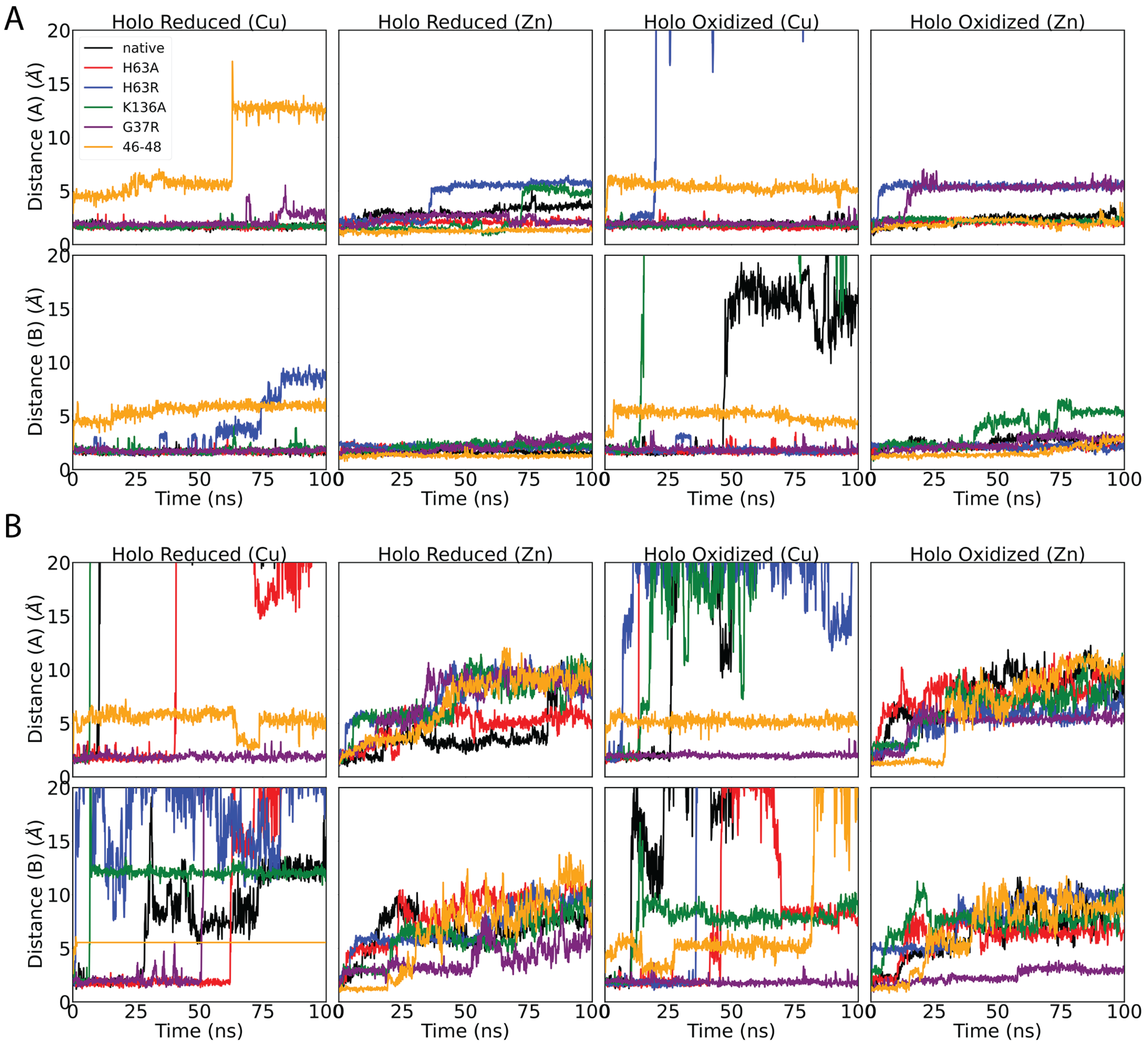

Figure S3. Metal coordination distance traced by averaging all the Cβ-metal distances for each metal **(A)** at 298 K and **(B)** at 400 K. Top panels show the chain A and bottom panels show the chain B. Wild-type-black, H63A-red, H63R-blue, K136A-green, G37R-purple, H46R/H48D-yellow.
